# Project management lessons learned from the multicentre CYCLE pilot randomized controlled trial

**DOI:** 10.1186/s13063-019-3634-7

**Published:** 2019-08-28

**Authors:** Devin S. McCaskell, Alexander J. Molloy, Laura Childerhose, F. Aileen Costigan, Julie C. Reid, Magda McCaughan, France Clarke, Deborah J. Cook, Jill C. Rudkowski, Christopher Farley, Tim Karachi, Bram Rochwerg, Anastasia Newman, Alison Fox-Robichaud, Margaret S. Herridge, Vincent Lo, Deanna Feltracco, Karen Burns, Rebecca Porteous, Andrew J. E. Seely, Ian M. Ball, Amy Seczek, Michelle E. Kho

**Affiliations:** 1 The Research Institute of St. Joe’s Hamilton, 50 Charlton Ave E, Hamilton, ON L8N 4A6 Canada; 2 McMaster University, School of Rehabilitation Science, Institute of Applied Health Science, Room 403, 1400 Main Street West, Hamilton, ON L8S 1C7 Canada; 3 Department of Health Research Methods, Evidence and Impact McMaster University Health Sciences Centre, Room 2C16, 1280 Main St W, Hamilton, ON L8S 4K1 Canada; 4 Department of Physiotherapy, St. Joseph’s Healthcare Hamilton, 50 Charlton Ave E, Hamilton, ON L8N 4A6 Canada; 5 Department of Medicine, McMaster University, 1280 Main St W, Hamilton, ON L8S 4L8 Canada; 6 Department of Critical Care, St. Joseph’s Healthcare Hamilton, 50 Charlton Ave E, Hamilton, ON L8N 4A6 Canada; 7 Juravinski Hospital, Hamilton, 711 Concession St, Hamilton, ON L8V 1C3 Canada; 8 Hamilton General Hospital, 237 Barton St E, Hamilton, ON L8L 2X2 Canada; 9 Toronto General Hospital, 200 Elizabeth St, Toronto, ON M5G 2C4 Canada; 10 Department of Physical Therapy, University of Toronto, Rehabilitation Sciences Centre, 8th Floor, 500 University Ave, Toronto, ON M5G 1V7 Canada; 11 St. Michael’s Hospital, 30 Bond St, Toronto, ON M5B 1W8 Canada; 12 The Ottawa Hospital, 501 Smyth Rd, Ottawa, ON K1H 8L6 Canada; 13 Department of Medicine and Department of Epidemiology and Biostatistics, Western University, Critical Care Trauma Centre, London Health Sciences Centre, 800 Commissioners Rd E, London, ON N6A 5W9 Canada

**Keywords:** Randomized controlled trial, Project management, Trial management, Critical care, Rehabilitation

## Abstract

**Background:**

Clinical trials management can be studied using project management theory. The CYCLE pilot randomized controlled trial (RCT) was conducted to determine the feasibility of a future rehabilitation trial of early in-bed cycling in the intensive care unit (ICU). In-bed cycling is a novel intervention, not typically available in ICUs. Implementation of this intervention requires personnel with specialized clinical expertise caring for critically ill patients and use of the in-bed cycle. Our objective was to describe the implementation and conduct of our pilot RCT using a project management approach.

**Methods:**

We retrospectively reviewed activities, timelines, and personnel involved in the trial. We organized activities into four project management phases: initiation, planning, execution, and monitoring and controlling. Data sources included Methods Centre documents used for trial coordination and conduct, and the trial data set. We report descriptive statistics as counts and proportions and also medians and quartiles, and we summarize the lessons learned.

**Results:**

Seven ICUs in Canada participated in the trial. Time from research ethics board and contracts submission to first enrolment was a median (first quartile, third quartile) of 185 (146, 209) and 162 (114, 181) days, respectively. We trained 128 personnel on the CYCLE pilot RCT protocol, and 80 (63%) completed trial-related activities. Four sites required additional training after start-up due to staff turnover and leaves of absence. Over 15 months, we screened 864 patients: 256 were eligible and 66 were enrolled. Despite an 85% consent rate, 74% (190/256) of eligible patients were not randomized, largely (80% [152/190]) due to physiotherapist availability. Thirteen percent of recruitment weeks were lost due to physiotherapist staffing shortages. We highlight five key lessons learned: (1) prepare and anticipate site needs; (2) communicate regularly; (3) proactively analyse and act on process measure data; (4) develop contingency plans; (5) express appreciation to participating sites.

**Conclusions:**

Our analysis highlights the scope of relevant activities, rigorous training and monitoring, number and types of required personnel, and time required to conduct a multicentre ICU rehabilitation intervention trial. Our lessons learned can help others interested in implementing complex intervention trials, such as rehabilitation.

**Trial registration:**

ClinicalTrials.gov, NCT02377830. Registered prospectively on 4 March 2015.

**Electronic supplementary material:**

The online version of this article (10.1186/s13063-019-3634-7) contains supplementary material, which is available to authorized users.

## Background

Randomized controlled trials (RCTs) are the reference standard for investigating the efficacy of an intervention, but they are challenging to conduct [1]. Many RCTs fail to start up, recruit their target sample, collect timely high-quality data, or adhere to their budget [2–4]. A review of 73 multicentre RCTs summarizing interventions in various clinical areas (e.g. cancer, mental health, orthopaedics) and settings (e.g. hospitals, general practice, community, mixed) found that only 55% recruited their target sample and 45% required an extension of their recruitment time [2]. Another review of 114 multicentre RCTs investigating assorted interventions (e.g. drugs, behavioural therapies, surgical procedures) found that fewer than one-third successfully recruited their target sample size within the planned timeframe [4]. Evidence-based guidance regarding best trial management practices and lessons learned from conducting RCTs are rarely shared amongst trialists [4–6].

Using project management theory, a clinical trial can be divided into five essential phases [5, 7, 8]. *Initiation* includes defining the project and obtaining authorization to start [5, 7]. *Planning* includes establishing the scope of the project, defining its objectives, and determining the course of action [7, 8]. *Execution* encompasses activities to complete the project requirements, including allocating resources and supporting trial team members to ensure their delegated activities are completed [7, 8]. *Monitoring and controlling* occurs simultaneously with execution. This phase involves tracking, reviewing, and regulating the project, identifying areas where changes to the project plan are needed, and initiating the changes [7, 8]. Lastly, *analysis and reporting* entails developing the final report, publishing results, and formally closing the trial [7].

Rehabilitation interventions for critically ill patients in the intensive care unit (ICU) are an emerging area of investigation. Survivors of critical illness are at significant risk for developing long-term physical disability, and early physical rehabilitation interventions may improve their functional outcomes [9–11]. However, ICU rehabilitation trials are challenging to implement, with published reports highlighting difficulties in recruiting their pre-specified target sample within time and budget constraints [12–15]. Given the complexity of physical rehabilitation interventions evaluated in trials of the critically ill and the multidisciplinary personnel involved, strategies to successfully conduct rigorous trials in this field are needed.

In-bed cycling is a novel, promising rehabilitation intervention for critically ill patients. The CYCLE pilot RCT (NCT02377830) was conducted in seven Canadian adult ICUs to assess the feasibility of in-bed cycling started within the first 4 days of mechanical ventilation and to inform the conduct of a larger future RCT [16, 17]. The objectives of this paper were to report the activities, timelines, and personnel involved in the CYCLE pilot RCT and highlight five lessons learned across four project management phases: initiation, planning, execution, and monitoring and controlling. Details regarding the analysis and reporting phase are published elsewhere [17].

## Methods

### CYCLE pilot RCT overview

The detailed trial protocol is published elsewhere [16]. Briefly, we randomized participants to two interventions: either Cycling, 30 min per day of in-bed cycling plus routine physiotherapy interventions, or Routine, routine physiotherapy interventions alone. We assessed participants’ physical function at ICU awakening, ICU discharge, and hospital discharge. Assessors, blinded to treatment intervention, conducted strength and function outcome measures at hospital discharge. Our five physical performance measures included the Physical Function ICU Test-scored (PFIT-s) [18–24], which was the anticipated primary outcome for the full RCT. We planned to enrol 60 patients in this pilot RCT [16].

**Fig. 1:**
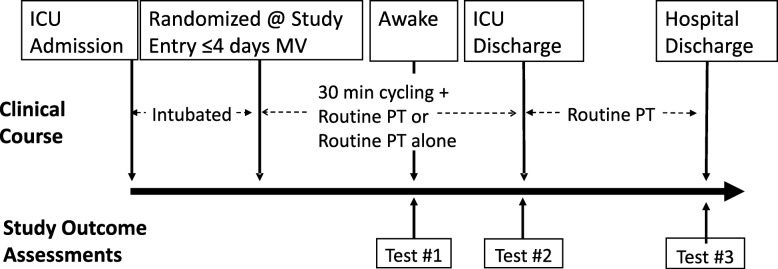
CYCLE pilot RCT schema. Participants were randomized within the first 4 days of mechanical ventilation (*MV*) to either 30 min of cycling plus routine physiotherapy or routine physiotherapy alone. Physical performance measures were collected at 3 time points: ICU awakening (Test #1), ICU discharge (Test #2), and hospital discharge (Test #3). Physical performance measures included manual muscle testing, the 30-s sit-to-stand test, the 2-min walk test, quadriceps femoris strength measured with a hand-held dynamometer, and the PFIT-s. The PFIT-s at hospital discharge was the primary outcome

The CYCLE Methods Centre at St. Joseph’s Healthcare Hamilton coordinated and managed all trial activities and data. Our Methods Centre personnel included the study principal investigator, a full-time research analyst (37.5 h/week), a part-time research coordinator (15 h/week), and a part-time research assistant (20 h/week). Site personnel included site principal investigators, ICU physiotherapists who delivered the cycling intervention, hospital physiotherapists and physical/occupational therapy assistants who collected physical performance measures, and research coordinators and research assistants who recruited participants, collected participant-reported outcomes and other data, and/or conducted data entry. The research coordinators obtained informed consent from the patients or their substitute decision makers prior to randomization. All seven participating sites were adult medical-surgical ICUs in academic hospitals in Ontario and active members of the Canadian Critical Care Trials Group. Figure 1 summarizes the trial schema.

### Project management review

#### Data sources

We retrospectively reviewed trial activities, timelines to study milestones, and the roles of trial personnel corresponding with project management phases. Data sources for all phases included Methods Centre documentation for trial coordination (e.g. principal investigator and research coordinator notes, email correspondence, meeting agendas and attendance sheets, expense reports, data entry and validation reports), trial conduct by site (e.g. research ethics board [REB] correspondence, delegation of authority logs, screening logs, randomization reports), and the trial data set. Initiation phase data also included trial registration records (i.e. ClinicalTrials.gov), grant applications, and funding documents.

We organized trial activities by project management phase [5]. *Initiation* included activities to activate the first site including grant submissions, REB and contracts submission, and trial registration. This phase ended upon enrolment of the first trial participant. *Planning* included activities by the Methods Centre to start the next six sites: individual REB applications, contracts negotiation, in-bed cycle ergometer procurement, creation of training materials, and development of case report forms and study database. We obtained ethics approval at each site because there was no central REB. On-site training sessions included three components: use of in-bed cycle ergometer with critically ill patients, conduct of strength and function outcome measures, and review of the protocol. This phase ended when all sites had enrolled at least one participant. *Execution* encompassed recruitment (e.g. screening patients for eligibility, obtaining informed consent, randomization), protocol delivery, data collection and entry, and data cleaning. It lasted until the data were fully cleaned at all sites. *Monitoring and controlling* overlapped with *Execution*. It included oversight of trial activities (e.g. recruitment, intervention fidelity, outcome collection, data entry and cleaning), weekly Methods Centre meetings and communication with site personnel, and additional training sessions delivered after site activation (e.g. refreshers and new staff). Table 1 lists the outcome measures in each of the four included phases. The *analysis and reporting* phase is excluded from this paper, as the results of the CYCLE pilot RCT are reported elsewhere [17].

**Table 1:** Outcome measures by project management phases

Project management phase	Project outcome measures
Initiation	• Time from REB submission to approval
	• Grant success rate
	• Time to first enrolment
Planning	• Time from site REB submission to approval
	• Time from site contracts submissions to approval
	• Number of and types of personnel trained
	• Number of training sessions
	• Materials prepared by the Methods Centre
	• Time to first enrolment
Execution	• Consent rate overall
	• Enrolment rate per month per site
	• Proportion of trained personnel who performed trial activities
	• Number of case report forms completed
	• Data query rate
	• Time to clean data
Monitoring and controlling	• Intervention fidelity by site
	• Intervention delivery (e.g. cycling)
	• Primary outcome measure collection (e.g. PFIT-s)
	• Number of recruitment weeks lost and reasons by site
	• Number of patients screened per 1 participant enrolled by site
	• Number of eligible non-randomized patients and reasons
	• Number of in-person training sessions required after start-up

### Statistical analysis

We conducted a descriptive analysis. We report binary data using counts and proportions, and continuous data using medians and interquartile range.

## Results

**Fig. 2:**
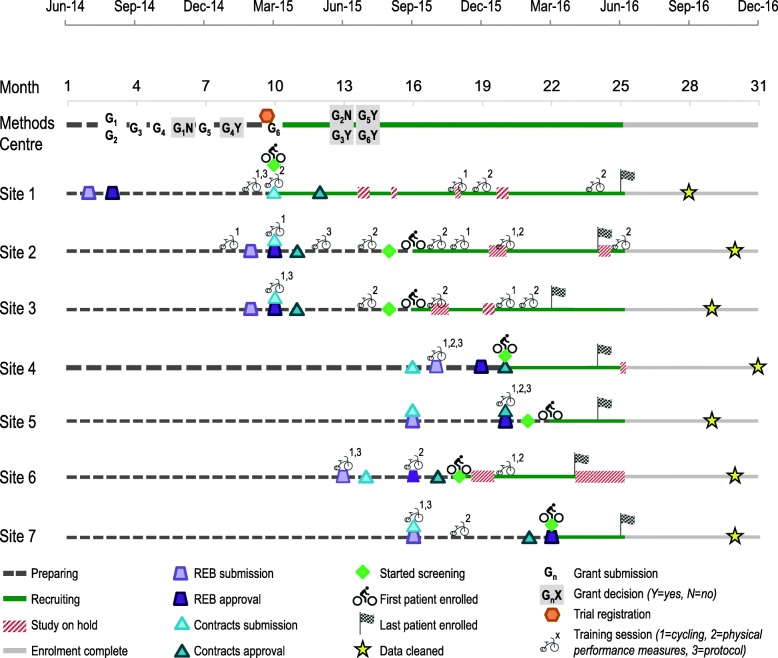
Timeline of CYCLE pilot RCT. Activities completed by the Methods Centre and participating sites from initial REB submission until the data were cleaned are summarized. Key milestones included receipt of funding, ethics and contracts approval, patient enrolment, and data cleaned. Training sessions included cycling intervention, physical performance measures, and/or protocol training. One of the Site 2 training sessions occurred after enrolling their last participant because we anticipated additional opportunities for recruitment

We randomized 66 patients in seven Ontario ICUs from March 2015 to June 2016 [17]. We initially planned to recruit 60 participants; we subsequently increased recruitment to 66 to account for cycling participants who did not receive the intervention or missed primary outcome assessments. The Methods Centre personnel provided on-site training at start-up and throughout the trial as needed, accounting for approximately 25% of the total study budget. Figure 2 and Additional file 1: Table S1 summarize the timelines of initiation, planning, and execution activities completed during the trial. We originally projected that we would recruit 60 patients by December 2017.

### Initiation

The Methods Centre initiated the study at Site 1 with a REB submission in July 2014 because our first grant proposal required ethics approval. The lead principal investigator submitted six grant applications between August 2014 and March 2015 and received four (67% success) see (Fig. 2). Whilst awaiting funding, we developed the case report forms, which were later used to build the database. We received notification of our first successful grant in January 2015 see (Fig. 2), which funded four sites enrolling patients > 65 years old. Following receipt of funding from other agencies, we opened additional sites and enrolled patients ≤ 65 years old at our first four sites. The CYCLE pilot RCT was registered with ClinicalTrials.gov on March 4, 2015 and the first participant was enrolled on March 25, 2015. By conducting this preparatory work during the initiation phase, we learned how to quickly initiate enrolment within 3 months at our first site and develop strategic plans for start-up at subsequent sites.

### Planning

#### Methods Centre activities

The Methods Centre supplied sites with materials for REB submissions (e.g. study protocol, informed consent form template, budget) and contracts. Timelines related to REB approval, contracts, and first enrolment were variable across sites (Fig. 2; Additional file 1: Table S1). Time from REB and contracts submissions to first enrolment was a median (first quartile [Q1], third quartile [Q3]) of 185 (146,209) and 162 (114,181) days, respectively. The Methods Centre trained 128 personnel to perform trial activities, preparing 164 training and seven regulatory binders (Additional file 1: Table S1). The Methods Centre revised case report forms throughout the study based on feedback from the team and launched the database in February 2016.

#### Site activities

The Methods Centre delivered start-up training sessions at the sites between January 2015 and January 2016. Sites 2 through 7 had significant experience conducting critical care trials; however, all physiotherapists and all sites were new to both in-bed cycling and ICU rehabilitation research. Training in trial coordination and data entry occurred in parallel with in-person protocol training as well as informally via telephone throughout the trial. During trial start-up sessions, the Methods Centre facilitated discussion amongst the site principal investigator(s), ICU physiotherapists, blinded outcomes assessors, and research coordinators regarding roles within the trial. Each site developed a plan to conduct cycling and outcome measures, track patients, and ensure a blinded assessor conducted physical function hospital discharge assessments (Fig. 1). From our planning activities, we learned that each site tailored the trial activities based on the experience and expertise of their own local research personnel.

### Execution

Of the 128 personnel trained in study components, 80 (63%) performed activities during the trial (Additional file 1: Table S1). Participating site characteristics and recruitment data are summarized in Additional file 1: Table S2.

#### Screening and enrolment

The seven sites screened a total of 864 patients: 608 (70%) were excluded, 256 (30%) were eligible, and 66 (8%) were enrolled. Our consent rate was 85% (66/78). We enrolled a median (Q1, Q3) of 1.1 (0.9, 1.3) participants/month/site whilst actively screening.

#### Cycling delivery

Of the ICU physiotherapists trained, 58% (21/36) performed at least one cycling session during the trial. Of the 15 physiotherapists who did not perform cycling, primary reasons included six who did not typically lead rehabilitation sessions in the ICU and three whose caseload did not include patients randomized to cycling. Some centres designated one therapist to provide the cycling intervention. Of the participants randomized to the cycling arm, 94% (34/36) received at least one cycling session. Participants cycled on 79% (146/184) of eligible days, with a median (Q1, Q3) delivery of 88% (67, 100) per participant, and 79% (115/146) of cycling sessions reached the target 30-min duration.

#### Physical performance measures

Of the clinicians (physiotherapists, physiotherapist assistants/occupational therapist assistants) trained in physical performance measures, 55% (32/58) performed at least one assessment. Similar to implementation of the cycling intervention, some sites designated one person to collect outcomes. We focused our analysis of physical performance measures on the blinded PFIT-s assessments at hospital discharge, as this was the anticipated primary outcome for a future large RCT. We collected 96% (43/45) of PFIT-s assessments from participants alive at hospital discharge, and 86% (37/43) were blinded. Additional file 1: Table S3 summarizes PFIT-s collection [6].

#### Data entry and cleaning

Of the personnel trained (research coordinators and research assistants; varied by site), 74% (14/19) performed data entry. In total, the study produced 4318 case report form pages. The Methods Centre sent 2248 queries during the data cleaning process, for a data query rate of 0.52 query per page. Site research coordinators and research assistants resolved all (100%) queries. Time from the last participant enrolled to data > 98% cleaned was a median (Q1, Q3) of 176 (150, 200) days (Additional file 1: Table S1). All data were cleaned by December 2016 (Fig. 2; Additional file 1: Table S1). During the execution phase, we learned that fewer people conducted their roles compared to those trained and that our study was acceptable in other centres (84% overall consent rate), and we identified opportunities for additional enrolment (74% [190/256] eligible not randomized).

### Monitoring and controlling

This phase overlapped temporally with execution.

#### Screening and enrolment

**Fig. 3:**
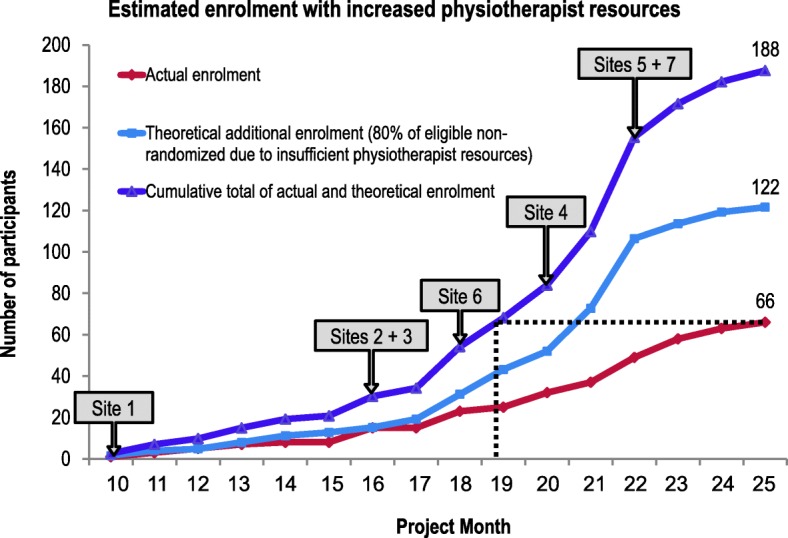
Actual and theoretical enrolment with increased physiotherapist resources in CYCLE pilot RCT. Theoretical additional enrolment was calculated assuming an 80% consent rate for all eligible non-randomized patients due to insufficient physiotherapist resources or no physiotherapist available. Site labels indicate the month during which the respective sites enrolled their first participant

The Methods Centre actively analysed screening and enrolment data continuously throughout the trial. We recruited the revised sample size 18 months earlier than anticipated. However, a total of 31 (13%) recruitment weeks were lost across five sites due to staffing shortages from physiotherapist turnover, vacations, and leaves of absence (Additional file 1: Table S2). We screened a median (Q1, Q3) of 12 [11, 15] patients for every participant enrolled. Seventy-four percent (190/256) of eligible patients were not randomized. Eighty percent (152/190) of eligible non-randomized patients were due to insufficient physiotherapist resources. Figure 3 models theoretical enrolment if we had approached patients classified as eligible non-randomized due to lack of physiotherapist resources and achieved an 80% consent rate. We estimate that recruitment could have been completed 6 months earlier with only four sites and sufficient physiotherapist staffing (Fig. 3). We estimate that we could also have enrolled 122 more participants over the study’s 15-month course at all seven sites if sufficient physiotherapist resources were available to enrol patients (Fig. 3).

#### Cycling delivery

During weekly communications, the Methods Centre and site research coordinators discussed cycling delivery and associated challenges. Cycling did not occur on 21% of eligible days. Physiotherapist workload accounted for missed cycling delivery on 10% of eligible days. Other reasons included patient decline, other patient activity prioritized, and family declined. Additional details regarding eligible days where cycling did not occur are reported elsewhere [17].

#### Physical performance measures collection

During weekly contact, site research coordinators and the Methods Centre discussed the clinical course of participants, estimated timelines for physical performance assessments, and planned to ensure an appropriate assessor would be available when needed. As a result, we missed only 4% (6/147) of PFIT-s assessments see (Additional file 1: Table S3).

#### Data entry and cleaning

The Methods Centre generated data entry and validation reports weekly, which were discussed at internal meetings. The Methods Centre performed all data management activities (e.g. data validation, issuing and resolving queries). Weekly correspondence with sites included reminders to enter data and respond to queries.

#### Additional activities and training after start-up

During the trial, we sent training certificates to participants who completed in-bed cycling or outcome measures to add to their professional portfolios. We also sent small tokens of appreciation ($5 coffee card) to site personnel during National Physiotherapy Month to recognize each centre’s contributions. As a result of regular communication with the sites, the Methods Centre determined that additional training sessions were required in four sites during the trial after enrolling their first participant (Fig. 2; Additional file 1: Table S1). In these sessions, the Methods Centre trained more personnel so at least one person would be available to perform essential trial activities. For example, Site 2 required multiple training sessions throughout the trial due to physiotherapists changing professional roles, leaves of absence, and new physiotherapists joining the site (Fig. 2). Regular communication during the monitoring and controlling phase helped us learn about the feasibility of the trial at other sites (79% intervention delivery, 96% primary outcomes measured); however, future studies need to consider the impact of the trial on physiotherapists’ time (missed opportunities for enrolment and missed intervention delivery due to limited physiotherapist capacity) and also requirements for additional training due to staff turnover.

### Lessons learned

Based on our data and experiences, we summarize five lessons learned for investigators and research coordinators conducting multicentre rehabilitation trials: (1) prepare and anticipate site needs; (2) communicate regularly with participating sites; (3) proactively analyse and act on process measure data; (4) develop contingency plans; (5) express appreciation to participating sites. These lessons span the four project management stages described previously. Table 2 summarizes these lessons, the corresponding project management phases, and relevant exemplars from the CYCLE pilot RCT.

**Table 2:** Five trial management lessons learned from the CYCLE pilot RCT

Lessons learned	Relevant phase(s)	Examples from the CYCLE pilot RCT
1. Prepare and anticipate site needs	Initiation	Complete material preparation whilst waiting for approval to enrol participants. The Methods Centre prepared grant applications, case report forms, screening log templates, operations manuals, regulatory binders, training binders, training session slide decks, and other materials whilst applying for REB approval and negotiating institutional contracts at other sites. Preparatory work prevented later delays in the trial life cycle
	Planning	Submit documents for REB and contracts as soon as possible at each site. In multicentre clinical trials, individual sites will require varying amounts of time to reach milestones. Be prepared to support sites with efficient systems by providing sample REB documents (e.g. protocols, consent forms). Aid sites by addressing questions and revisions quickly and completing as much trial preparation as possible whilst awaiting approvals
	Planning	Select personnel with relevant experience and skill sets at each site to become team member early in the project life cycle
		We engaged ICUs through the Canadian Critical Care Trials Group, and the research coordinators at these sites had experience screening and obtaining consent from ICU patients’ and/or their substitute decision makers. In our trial, screening for eligibility, obtaining informed consent, and randomization all had to occur within 4 days, a relatively short period of time from ICU admission. Obtaining informed consent from ICU patients’ substitute decision makers can be challenging [25]. The patient’s substitute decision maker may be uncertain of the patient’s wishes regarding research participation, have a limited understanding of research in critical care, and be anxious about research participation [25]
		Training of frontline physical therapy clinicians comprised the majority of the CYCLE pilot RCT’s planning phase. We engaged physical therapy clinicians (physiotherapists, physiotherapist assistants/occupational therapist assistants) with experience assessing and treating similar patient populations to deliver the intervention and collect physical performance measures
	Planning	Tailor training to individual site needs and experience levels. In the CYCLE pilot RCT, the ICU physiotherapists at Site 1 had cycling experience from a previous trial [26]; thus, they only received start-up training in the protocol, physical performance measurements, and research coordination. At other sites, we provided training on cycling, outcome measures, and study processes. New sites had opportunities to practice cycling and collecting physical outcome measures for at least 1 month before enrolling their first participant
		Frontline clinician training should be planned sufficiently in advance to ensure that they are comfortable and competent with trial activities [5]. However, it should not occur so far in advance as to risk wasting time and financial resources on personnel who might not participate in the trial. We suggest prioritizing the training of key trial personnel (e.g. interventionists, research coordinators, lead outcomes assessors) who can act as trial champions. Trial champions can become trainers for new staff to optimize training efficiencies
	Execution	Start data management early in the trial life cycle and anticipate data cleaning for at least 6 months after completing data collection
2. Communicate regularly with participating sites	Monitoring and controlling	Regular personalized communication between the central management team and site personnel is essential. Weekly communication allowed site personnel to notify the Methods Centre about trial challenges, such as staffing issues. As a result, the Methods Centre trained new personnel to minimize effects on recruitment and protocol adherence. Maintaining communication and a professional but personal relationship with trial personnel is challenging and requires significant time, but results in a more successful trial [5]
3. Proactively analyse and act on process measure data	Monitoring and controlling	Use screening logs to monitor clinical trial recruitment. From our screening log data, we identified a high consent rate but also had a high eligible non-randomized rate. Most eligible non-randomized patients were related to ICU physiotherapist capacity, suggesting that continuous recruitment depended on the availability of these frontline clinicians. The Methods Centre responded to this need by training additional personnel. We trained physiotherapists from other areas of the hospital and physiotherapist assistants/occupational therapist assistants to conduct physical performance measures to try to reduce the extra clinical responsibility of ICU physiotherapists
4. Develop contingency plans	Monitoring and controlling	Anticipate losing at least 10% recruitment time lost due to staffing gaps. Although our trial met its target sample size earlier than expected, we lost 13% of potential recruitment weeks due to leaves of absence, vacation, and staff turnover. Eligible participants could not be approached for consent without physiotherapist capacity to enrol and conduct the intervention. Our results suggest that both the number and availability of frontline therapists was an important factor in rehabilitation trials
	Monitoring and controlling	Be prepared to train approximately one-third more trial personnel and allocate time and study budget to provide training sessions throughout the trial. We trained extra physiotherapists, physiotherapist assistants/occupational therapist assistants, research assistants, and research coordinators to replace personnel who changed roles and to account for staffing gaps
5. Express appreciation to participating sites	Execution	Express appreciation to frontline clinicians for their contributions. The CYCLE pilot RCT’s success relied on frontline clinicians dedicated to protocol fidelity for cycling and outcomes data. Clinicians added the trial activities to their usual workload. During the trial, we provided training certificates and recognized Canadian National Physiotherapy Month by offering therapists coffee gift cards. We acknowledged research coordinators, research assistants, ICU physiotherapists, and outcomes assessors in our publications. Recognizing the efforts of clinicians involved in the trial helps to ensure continued involvement and protocol adherence [5]

This table outlines the 5 key lessons learned from the CYCLE pilot RCT

*RCT* randomized controlled trial, *REB* research ethics board, *ICU* intensive care unit

## Discussion

The CYCLE pilot RCT finished recruitment early, met its recruitment target, and obtained high-quality data. We trained more than 128 research personnel, in seven different ICUs, and taught six centres how to implement and evaluate a novel rehabilitation technology in the complex critical care environment. Our analysis summarizes the activities and professionals involved in four project management phases of a multicentre ICU rehabilitation trial. We excluded the fifth project management phase, analysis and reporting, from this work because the results of the CYCLE pilot RCT are reported elsewhere [17]. We present five lessons learned that could help others planning to conduct their own multicentre rehabilitation trials.

Outcome measures are crucial to monitoring clinical trial progress. Based on our experience with the CYCLE pilot RCT, we propose tracking the following outcomes by project management phase:

- *Initiation*. Receipt of funding and initial ethics approval are most important, because without both, the trial cannot begin. Enrolment of the first patient is also a critical milestone.
- *Planning*. Document time to ethics and contracts approvals, identify the number of people required for training sessions, and record the time to first enrolment at each site.
- *Execution, Monitoring and controlling*. Throughout the trial, scrutinize the enrolment, reasons for screen failures, and eligible non-randomized patients. Monitor trial fidelity, including intervention delivery (cycling) and collection of primary outcomes (PFIT-s). Finally, monitor data entry, clean data, and track responses to data queries across all sites.

We believe that focusing on these outcome measures that describe the rigour and timeliness of process measures could be useful to trialists and trial personnel for managing these complex projects.

### Relationship to previous research

There is a lack of trial management research published in the literature. Arundel and Gellatly (2018) applied clinical trial management methods to the Obsessive Compulsive Treatment Efficacy Trial (OCTET) [8]. OCTET was a three-arm RCT that evaluated guided self-help, computerized cognitive behavioural therapy, and waiting for cognitive behavioural therapy in adults experiencing obsessive compulsive disorder symptoms [8]. Similar to our study, OCTET investigators trained specialized personnel, psychological wellbeing practitioners, to deliver their interventions [8]. In contrast to our study, OCTET occurred in the outpatient setting, and investigators used qualitative methods to evaluate the acceptability of clinical trials management methods during the execution and monitoring phases [8]. Both studies highlight the importance of communication, support provided by the management team to frontline clinicians, and tailored training to trainees’ experience level, offered frequently throughout the trial [8].

Both the OCTET and the CYCLE pilot RCT demonstrate that the project management framework can be applied to RCTs in different therapeutic areas, clinical settings, and patient populations. Our analysis advances the literature for evidence-informed trial management by reporting quantitative metrics that help describe and characterize four of five project phases. Below, we provide specific advice for trials of physical rehabilitation interventions in the ICU, which can also guide complex intervention studies.

Rehabilitation trials require specialized personnel with clinical expertise in the patient population under study. These trials are more time- and human resource-intensive than other types of interventions such as drug trials, because therapists are typically present for the duration of the intervention. A retrospective analysis of AVERT (A Very Early Rehabilitation Trial for Stroke) similarly reported a 10% loss in time to recruit their target sample size due to personnel parental leaves; it also reported that it took longer than initially planned to complete recruitment [12]. Evaluation of a new technology requires training and time for personnel to become familiar with the technology before applying it in a trial. Given these requirements, investigators planning rehabilitation trials or complex interventions need to consider contingency plans to ensure sufficient availability of specialized personnel with the clinical expertise to deliver the trial intervention.

Our study has limitations. We conducted a retrospective analysis using available documents that were not originally developed for this purpose. For example, we did not document the time spent creating materials, preparing for training sessions, or preparing for meetings, which underestimates the time associated with the planning phase. We also did not collect the amount of time spent on data entry at each site. Our Methods Centre personnel estimates may not be generalizable to other studies; however, we outline key roles and responsibilities of these critical staff which are likely applicable to many studies. Because this was a phase II pilot trial, we had a relatively small sample of seven participating sites, which may affect generalizability. Finally, our findings may have limited generalizability to trials conducted with different personnel or in settings unaccustomed to clinical research.

Our study also has several strengths. This analysis draws from practical experience to characterize and describe the activities, personnel, and time required to conduct a multicentre clinical research trial in rehabilitation. To our knowledge, we are the first to apply a project management approach to describing the conduct of a multicentre ICU rehabilitation trial. These results may help to inform the planning of realistic trial activities and milestones by methods centre and across participating sites for other multicentre rehabilitation trials. Given the complexity of multicentre trials, elements of our study, including documentation of time for ethics and contracts approvals, personnel training, and trial monitoring (fidelity and data cleaning), can inform other multicentre trials.

## Conclusions

Trial management for a multicentre ICU rehabilitation intervention requires rigorous training and monitoring. Our analysis of the CYCLE pilot RCT highlights the scope of relevant activities, diversity of personnel across sites, and amount of time required to conduct this type of trial. Our five key lessons learned included preparation and anticipation of site needs, regular communication with participating sites, proactive analysis and action on process measure data, development of contingency plans, and expressions of appreciation to participating sites. This study can inform the design and implementation of future complex interventions such as rehabilitation trials in the ICU.

## Additional file

Additional file 1: **Table S1.** Activities and milestones of the CYCLE pilot RCT’s initiation, planning, and execution phases. **Table S2.** CYCLE pilot RCT participating site characteristics and recruitment data. **Table S3.** PFIT-s collection data during the CYCLE pilot RCT. (DOCX 31 kb)

## Data Availability

The data sets used and/or analysed during the current study are available from the corresponding author on reasonable request and ethics approval.

## Abbreviations

ICU: Intensive care unit
PFIT-s: Physical Function ICU Test-scored
Q1: First quartile
Q3: Third quartile
RCT: Randomized controlled trial
REB: Research ethics board
