# Correction to: Project management lessons learned from the multicentre CYCLE pilot randomized controlled trial

**DOI:** 10.1186/s13063-019-3716-6

**Published:** 2019-10-25

**Authors:** Devin S. McCaskell, Alexander J. Molloy, Laura Childerhose, F. Aileen Costigan, Julie C. Reid, Magda McCaughan, France Clarke, Deborah J. Cook, Jill C. Rudkowski, Christopher Farley, Tim Karachi, Bram Rochwerg, Anastasia Newman, Alison Fox-Robichaud, Margaret S. Herridge, Vincent Lo, Deanna Feltracco, Karen E. A. Burns, Rebecca Porteous, Andrew J. E. Seely, Ian M. Ball, Amy Seczek, Michelle E. Kho

**Affiliations:** 1 The Research Institute of St. Joe’s Hamilton, 50 Charlton Ave E, Hamilton, ON L8N 4A6 Canada; 2 McMaster University, School of Rehabilitation Science, Institute of Applied Health Science, Room 403, 1400 Main Street West, Hamilton, ON L8S 1C7 Canada; 3 Department of Health Research Methods, Evidence and Impact McMaster University Health Sciences Centre, Room 2C16, 1280 Main St W, Hamilton, ON L8S 4K1 Canada; 4 Department of Physiotherapy, St. Joseph’s Healthcare Hamilton, 50 Charlton Ave E, Hamilton, ON L8N 4A6 Canada; 5 Department of Medicine, McMaster University, 1280 Main St W, Hamilton, ON L8S 4L8 Canada; 6 Department of Critical Care, St. Joseph’s Healthcare Hamilton, 50 Charlton Ave E, Hamilton, ON L8N 4A6 Canada; 7 Juravinski Hospital, Hamilton, 711 Concession St, Hamilton, ON L8V 1C3 Canada; 8 Hamilton General Hospital, 237 Barton St E, Hamilton, ON L8L 2X2 Canada; 9 Toronto General Hospital, 200 Elizabeth St, Toronto, ON M5G 2C4 Canada; 10 Department of Physical Therapy, University of Toronto, Rehabilitation Sciences Centre, 8th Floor, 500 University Ave, Toronto, ON M5G 1V7 Canada; 11 St. Michael’s Hospital, 30 Bond St, Toronto, ON M5B 1W8 Canada; 12 The Ottawa Hospital, 501 Smyth Rd, Ottawa, ON K1H 8L6 Canada; 13 Department of Medicine and Department of Epidemiology and Biostatistics, Western University, Critical Care Trauma Centre, London Health Sciences Centre, 800 Commissioners Rd E, London, ON N6A 5W9 Canada

**Correction to: Trials (2019) 20: 532**

**https://doi.org/10.1186/s13063-019-3634-7**

Following publication of the original article [1], we have been notified that one of the authors’ names is spelled incorrectly. In this Correction the incorrect and correct author name are shown.

Originally the author name has been published as:

- Karen Burns

The correct author name is:

- Karen EA Burns

The original article has been corrected.
