# Supplementary material for: Project management lessons learned from the multicentre CYCLE pilot randomized controlled trial
Source: Trials. 2019 Aug 28;20:532. doi: 10.1186/s13063-019-3634-7 (PMC6712681; doi:10.1186/s13063-019-3634-7)
Supplement: Supplementary file 1 — Table S1. Activities and milestones of the CYCLE pilot RCT’s initiation, planning, and execution phases. Table S2. CYCLE pilot RCT participating site characteristics and recruitment data. Table S3. PFIT-s collection data during the CYCLE pilot RCT. (DOCX 31 kb) [file 13063_2019_3634_MOESM1_ESM.docx]

**Additional file**

**Table S1.** **Activities and milestones of the CYCLE Pilot RC’s initiation, planning, and execution phases.** Personnel from Sites 1, 2, 3, and 6 participated in multi-site cycling and physical performance measures training sessions. Data entry activities for Sites 1 and 3 were conducted by multi-site data entry personnel.

|  | | Site 1 | Site 2 | Site 3 | Site 4 | Site 5 | Site 6 | Site 7 | Multi-site | Total | Median (Q1,Q3) |
| --- | --- | --- | --- | --- | --- | --- | --- | --- | --- | --- | --- |
| Time to milestones (days) | REB submission to approval | 34 | 21 | 21 | 69 | 110 | 95 | 176 |  |  | 69 (28,103) |
|  | REB submission to first enrolment | 260 | 205 | 213 | 95 | 161 | 130 | 185 |  |  | 185 (146,209) |
|  | REB approval to first screen | 224 | 146 | 146 | 19 | 49 | 34 | 6 |  |  | 49 (27,146) |
|  | REB approval to first enrolment | 226 | 184 | 192 | 26 | 51 | 35 | 9 |  |  | 51 (31,188) |
|  | Contracts submission to approval | 48 | 23 | 36 | 126 | 120 | 70 | 156 |  |  | 70 (42,123) |
|  | Contracts submission to first enrolment | 1 | 177 | 185 | 131 | 162 | 97 | 197 |  |  | 162 (114,181) |
|  | Contracts approval to first screen | -49 | 116 | 103 | -2 | 40 | 26 | 38 |  |  | 38 (12,72) |
|  | Contracts approval to first enrolment | -47 | 154 | 149 | 5 | 42 | 27 | 41 |  |  | 41 (16,96) |
|  | First to last start-up training session | 23 | 196 | 118 | 6 | 1 | 83 | 42 |  |  | 42 (15,101) |
|  | Start-up training completion to first screen | 18 | 12 | 18 | 102 | 47 | 48 | 144 |  |  | 47 (18,75) |
|  | First screen to first enrolment | 2 | 38 | 46 | 7 | 2 | 1 | 3 |  |  | 3 (2,23) |
|  | Start-up training completion to first enrolment | 18 | 12 | 18 | 102 | 47 | 48 | 144 |  |  | 47 (18,75) |
|  | Last enrolment to data cleaned | 110 | 176 | 196 | 203 | 146 | 223 | 153 |  |  | 176 (150,200) |
| Training sessions per site (n) | Cycling | 1 | 4 | 2 | 1 | 1 | 1 | 1 | 2 | 13 | 1 (1,2) |
|  | Physical performance measures | 2 | 1 | 2 | 1 | 1 | 1 | 1 | 3 | 12 | 1 (1,2) |
|  | Research coordination | 1 | 1 | 1 | 1 | 1 | 1 | 1 | 0 | 7 | 1 (1,1) |
| Training time per site (hours) | Cycling | 5.0 | 12.0 | 8.0 | 7.0 | 6.0 | 6.0 | 7.0 | 8.0 | 59.0 | 7.0 (6.0,7.5) |
|  | Physical performance measures | 2.0 | 1.0 | 4.0 | 3.3 | 3.3 | 2.7 | 2.0 | 8.0 | 26.3 | 2.7 (2.0,3.5) |
|  | RC assessments | 2.0 | 1.0 | 1.0 | 1.5 | 2.0 | 1.0 | 2.0 | 0.0 | 10.5 | 1.5 (1.0,2.0) |
| Personnel conducted / Personnel trained (n) | Cycling | 4/6 | 5/6 | 4/6 | 2/3 | 2/2 | 3/9 | 1/4 | 0/0 | 21/36 | 3 (2,4) / 6 (4,6) |
|  | Physical performance measures | 9/13 | 3/10 | 5/10 | 4/7 | 2/4 | 6/9 | 3/5 | 0/0 | 32/58 | 4 (3,6) / 9 (6,10) |
|  | RC assessments | 2/2 | 1/1 | 1/1 | 2/3 | 1/2 | 4/4 | 2/2 | 0/0 | 13/15 | 2 (1,2) / 2 (2,3) |
|  | Data entry | 0/2 | 1/1 | 0/1 | 2/3 | 2/3 | 4/4 | 2/2 | 3/3 | 14/19 | 2 (1,2) / 2 (2,3) |
| Training materials created (n) | Cycling | 12 | 9 | 8 | 8 | 9 | 14 | 7 |  | 67 | 9 (8,11) |
|  | Physical performance measures | 16 | 13 | 12 | 12 | 9 | 14 | 8 |  | 84 | 12 (11,14) |
|  | Research coordination | 2 | 1 | 1 | 3 | 2 | 2 | 2 |  | 13 | 2 (2,2) |

**Table S2. CYCLE Pilot RCT participating site characteristics and recruitment data.** Department-based (“Department”) PT organizational structure is defined as the hospital having one central PT department where the PTs are shared throughout the hospital. Program management-based (“Program”) PT organizational structure is defined as each unit and ward within the hospital employs their own PTs.

| Site Characteristics | Site 1 | Site 2 | Site 3 | Site 4 | Site 5 | Site 6 | Site 7 | Overall | Median (Q1,Q3) |
| --- | --- | --- | --- | --- | --- | --- | --- | --- | --- |
| Location  (City, Province) | Hamilton, ON | Hamilton, ON | Hamilton, ON | Toronto, ON | Ottawa,  ON | Toronto, ON | London,  ON |  |  |
| PT organizational structure | Department | Program | Program | Program | Program | Program | Program |  |  |
| Total weeks on study (n) | 66 | 41 | 40 | 22 | 17 | 34 | 13 | 233 | 34 (20,41) |
| Total active screening time (weeks) | 60 | 40 | 35 | 21 | 17 | 22 | 13 | 208 | 22 (19,38) |
| Screening or randomization on hold (weeks) | 6 | 5 | 5 | 1 | 0 | 14 | 0 | 31 | 5 (1,6) |
| Average enrolment per active screening week (n) | 0.37 | 0.25 | 0.23 | 0.43 | 0.13 | 0.41 | 0.38 | 2.20 | 0.37 (0.24,0.40) |
| Patients screened per one patient randomized (n) | 11 | 12 | 25 | 5 | 13 | 18 | 12 | 13 | 12 (11,15) |
| Patients randomized (n) | 22 | 10 | 8 | 9 | 3 | 9 | 5 | 66 | 9 (7,10) |

**Table S3.** **PFIT-s collection data during the CYCLE Pilot RCT.** PFIT-s was collected at 3 time points: ICU awakening, ICU discharge, and hospital discharge. PFIT-s score at hospital discharge, collected by a blinded assessor, was anticipated to be the primary outcome for the future large RCT of in-bed cycling. Participants are eligible for an ICU awakening assessment when they pass cognitive screening and are able to follow commands. Participants are eligible for ICU and hospital discharge assessments once the respective discharge orders are written.

|  | ICU Awakening | ICU Discharge | Hospital Discharge |
| --- | --- | --- | --- |
| Randomized (n) | 66 | 66 | 66 |
| Alive at time point and eligible for assessment (n (%)) | 52 (79%) | 50 (76%) | 45 (68%) |
| PFIT-s assessments not completed (n (%)) | 0 (0%) | 4 (8%) | 2 (4%) |
| *Assessor perceives patient unable to perform (n) | 0 | 2 | 0 |
| Patient refusal (n) | 0 | 1 | 0 |
| Missed (n) | 0 | 1 | 2 |
| PFIT-s assessments completed (n (%)) | 52 (100%) | 46 (92%) | 43 (96%) |
| PFIT-s assessments collected after hospital discharge (n) |  |  | 3 |
| PFIT-s assessments conducted by blinded assessor (n (%)) |  |  | 37 (86%) |

*Assessor Perceives Patient Unable to Perform Reasons (n=2): Suspected myocardial infarction and not appropriate for therapy (n=1); Patient edematous and not appropriate for therapy (n=1)
